# LRRK2 Phosphorylation, More Than an Epiphenomenon

**DOI:** 10.3389/fnins.2020.00527

**Published:** 2020-06-16

**Authors:** Antoine Marchand, Matthieu Drouyer, Alessia Sarchione, Marie-Christine Chartier-Harlin, Jean-Marc Taymans

**Affiliations:** ^1^University of Lille, Inserm, CHU Lille, U1172 - LilNCog - Lille Neuroscience & Cognition, Lille, France; ^2^Inserm, UMR-S 1172, Team “Brain Biology and Chemistry”, Lille, France

**Keywords:** LRRK2, phosphorylation, Parkinson's disease, kinase, phosphatase, phenotype

## Abstract

Mutations in the Leucine Rich Repeat Kinase 2 (LRRK2) gene are linked to autosomal dominant Parkinson's disease (PD), and genetic variations at the LRRK2 locus are associated with an increased risk for sporadic PD. This gene encodes a kinase that is physiologically multiphosphorylated, including clusters of both heterologous phosphorylation and autophosphorylation sites. Several pieces of evidence indicate that LRRK2's phosphorylation is important for its pathological and physiological functioning. These include a reduced LRRK2 heterologous phosphorylation in PD brains or after pharmacological inhibition of LRRK2 kinase activity as well as the appearance of subcellular LRRK2 accumulations when this protein is dephosphorylated at heterologous phosphosites. Nevertheless, the regulatory mechanisms governing LRRK2 phosphorylation levels and the cellular consequences of changes in LRRK2 phosphorylation remain incompletely understood. In this review, we present current knowledge on LRRK2 phosphorylation, LRRK2 phosphoregulation, and how LRRK2 phosphorylation changes affect cellular processes that may ultimately be linked to PD mechanisms.

## Introduction

Parkinson's disease (PD) is the most common motor neurodegenerative disease, and the gene encoding the protein Leucine Rich Repeat Kinase 2 (*LRRK2*) is considered one of the most important genetic risk factors for PD (Nalls et al., 2019). First, mutations in LRRK2 have been linked to autosomal dominant forms of PD and represent a relatively frequent genetic cause of PD, affecting 1 to 5% of PD patients (Paisán-Ruíz et al., 2008). The most common mutation is the LRRK2 G2019S, affecting up to 40% of patients in specific ethnicities from north African population (Healy et al., 2008; Lesage et al., 2010). In addition to causal mutations in the LRRK2 coding sequence, association studies and genome-wide association studies have revealed that other genetic variations at the LRRK2 locus modulate risk for sporadic PD (Satake et al., 2009; Simón-Sánchez et al., 2009; Ross et al., 2011; Nalls et al., 2019). Of interest, clinical phenotypes of PD patients carrying mutated forms of LRRK2 are very similar to the clinical manifestation in idiopathic PD, suggesting that LRRK2 may mediate pathogenic mechanisms relevant to all forms of PD (Marras et al., 2011; Langston et al., 2015).

**Figure 1:**
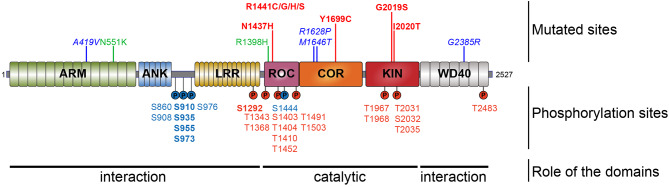
Schematic representation of LRRK2. Domain topology of LRRK2 is noted as well as their main feature (interaction or catalytic). Mutations segregating with the disease are indicated in the upper part and the phosphorylation sites are in the lower part. Pathogenic mutants are depicted in red, the risk variants are indicated in blue, and the two protective variants are in green. The heterologous phosphorylation sites are indicated in blue and the sites of autophosphorylation are in red. The most described and studied sites are indicated in bold.

*LRRK2* encodes a 286-kDa multi-domain protein of 2527 amino acids harboring two enzymatic activities, a kinase domain “KIN” and a GTPase domain named a Ras Of Complex proteins “ROC” (Figure 1). These two domains are connected via a C-terminal of ROC “COR” domain. This catalytic core is flanked by additional domains with predicted protein–protein interaction functions such as the leucine-rich repeat (LRR), Ankyrin repeat (ANK), and Armadillo repeat (ARM) domains at the N-terminus and the WD40 domain at the C-terminus (Mata et al., 2006; Cookson, 2010; Mills et al., 2012). Structurally, LRRK2 forms as a dimer under native conditions (Greggio et al., 2008). LRRK2 is a serine/threonine kinase of the tyrosine kinase-like family (Manning et al., 2002). Activation of serine/threonine kinases usually occurs by autophosphorylation of one or many residues in the activation loop. Such a phosphorylation can change the conformation associated with the ATP binding site and/or substrate interaction-binding site, resulting in the activation of the kinase. Evidence suggests that the N- and C-terminal regions of LRRK2 act as modulators of kinase activity or substrate specificity. Indeed, C-terminal truncation of the WD40 domain leads to the complete loss of kinase activity (Jorgensen et al., 2009), and deletion of N-terminal sequences of LRRK2 (LRRK2^970−2527^, LRRK2^1326−2527^) strongly reduces or abolishes the phosphorylation of LRRK2 substrates such as P62 and RAB7L1 (RAB29), but the phosphorylation of RAB10 and RAB10 is conserved, although autophosphorylation is maintained (Kalogeropulou et al., 2018).

Most *LRRK2* PD mutations are located in the catalytic core of the protein: in the ROC domain (N1437H, R1441C/G/H/S), in the COR domain (Y1699C), and in the kinase domain (G2019S, I2020T) (Funayama et al., 2005; Healy et al., 2008; Aasly et al., 2010; Mata et al., 2016; Nucifora et al., 2016). To date, low-resolution 3D structures of the homodimeric full-length LRRK2 have been reported by TEM and CRYO-EM (Guaitoli et al., 2016; Sejwal et al., 2017). More recently, higher-resolution structures of the C-terminal domains of LRRK2 coordinated around microtubules have been reported in BioRxiv (Watanabe et al., 2020). Several studies have reported altered dimerization for LRRK2 variants mutated in the ROC COR domain (Greggio et al., 2008; Klein et al., 2009; Daniëls et al., 2011; Memou et al., 2019), suggesting that LRRK2 disease mutations may alter the conformation of the LRRK2 dimer.

LRRK2 protein is expressed in a large variety of tissues. It is highly expressed in the lung, spleen, kidney, and immune cells (lymphocytes, monocytes, and neutrophils), while in the brain, there is a comparatively low level of LRRK2 expression (Paisán-Ruíz et al., 2004; Zimprich et al., 2004; Taymans et al., 2006; Kubo et al., 2010; Thévenet et al., 2011; Dzamko et al., 2013; West et al., 2014; Fan et al., 2018). It is also possible to detect LRRK2 in fluids such as urine, plasma, and cerebrospinal fluid (CSF) (reviewed in Taymans et al., 2017). In the cell, the protein is mainly cytoplasmic. LRRK2 is localized with an affinity for vesicles associated with microtubules, endoplasmic reticulum and Golgi apparatus, Trans-Golgi network (TGN), endosome, and lysosome (West et al., 2005, 2007; Biskup et al., 2006; Hatano et al., 2007; Sanna et al., 2012). LRRK2 is found to locate to mitochondrial outer membrane, membrane micro-domains such as the neck of caveolae, microvilli/filopodia, and intraluminal vesicles of multivesicular bodies identified by immunogold staining combined with electron microscopy (Alegre-Abarrategui et al., 2009). A proportion of LRRK2 puncta colocalizes with the proteins p62 and LC3 and a discrete colocalization with calnexin and frequently locate close to the gamma-tubulin positive centrosome.

The precise biological role of LRRK2 is not fully understood. However, to date, the protein has been shown to be involved in different cellular processes such as the regulation of cytoskeleton, neurite morphology, inflammatory processes, regulation of mitochondrial fission, protein synthesis, proteostasis, and vesicular trafficking (Esteves et al., 2014). The multitude of proposed functions can be summarized by findings from protein interaction network analysis that point to a role for LRRK2 in intracellular organization, intracellular transport, and protein localization (Manzoni et al., 2015; Porras et al., 2015; Tomkins et al., 2018).

## LRRK2 Phosphorylation

LRRK2 is a highly phosphorylatable protein. First, LRRK2 exists as a phosphorylated protein in mammalian cells under basal conditions as observed after metabolic labeling of LRRK2-expressing cells with radioactive phosphate or by detection of LRRK2 in phosphoprotein isolates from cell culture (Greggio et al., 2007; Lobbestael et al., 2013; Reyniers et al., 2014). Second, additional phosphorylation potential above the cellular phosphorylation of LRRK2 can be observed when purified LRRK2 is submitted to *in vitro* autophosphorylation (Reynolds et al., 2014). Third, in a similar fashion, *in vitro* incubation of LRRK2 with a separate kinase, such as protein kinase A (PKA), can also lead to additional phosphorylation of LRRK2 (Muda et al., 2014). The notion that LRRK2 is a highly phosphorylated protein is confirmed by phosphosite mapping studies via mass spectrometry, showing at least 74 phosphorylation sites on isolated LRRK2 protein, corresponding to almost 3% of all amino acid residues of the protein (Table 1) (Greggio et al., 2009; Kamikawaji et al., 2009; Gloeckner et al., 2010; Pungaliya et al., 2010). Phosphorylation sites include a majority of serines (59%), followed by 37% threonines and some tyrosines (4%). Further compilation of the reported LRRK2 phosphosites indicates that 37 are reported by two or more separate studies, meaning that half of the reported LRRK2 phosphorylation sites still await independent confirmation.

**Table 1:** Reported LRRK2 phosphorylation sites.

**Position**	**Number of paper**	**Techniques**	**Auto/heterologous phosphorylation site**	**Phospho Ab**	**Effect**
3 (Serine)	1 (Gloeckner et al., 2010)	*In vitro* autokinase assay followed by mass spectrometric analysis	Autophosphorylation (Gloeckner et al., 2010)	No	No effect described
5 (Serine)	1 (Gloeckner et al., 2010)	*In vitro* autokinase assay followed by mass spectrometric analysis	Autophosphorylation (Gloeckner et al., 2010)	No	No effect described
424 (Threonine)	1 (Gloeckner et al., 2010)	*In vitro* autokinase assay followed by mass spectrometric analysis	Autophosphorylation (Gloeckner et al., 2010)	No	No effect described
524 (threonine)	1 (Gloeckner et al., 2010)	*In vitro* autokinase assay followed by mass spectrometric analysis	Autophosphorylation (Gloeckner et al., 2010)	No	No effect described
776 (Threonine)	1 (Gloeckner et al., 2010)	*In vitro* autokinase assay followed by mass spectrometric analysis	Autophosphorylation (Gloeckner et al., 2010)	No	No effect described
826 (Threonine)	1 (Gloeckner et al., 2010)	*In vitro* autokinase assay followed by mass spectrometric analysis	Autophosphorylation (Gloeckner et al., 2010)	No	No effect described
833 (Threonine)	2 (Gloeckner et al., 2010; Muda et al., 2014)	*In vitro* autokinase assay followed by mass spectrometric *in vitro* analysis Phosphopeptide enrichment by PKA followed by mass spectrometric analysis	Autophosphorylation (Gloeckner et al., 2010) PKA phosphorylation (Muda et al., 2014)	No	No effect described
838 (Threonine)	1 (Gloeckner et al., 2010)	*In vitro* autokinase assay followed by mass spectrometric analysis	Autophosphorylation (Gloeckner et al., 2010)	No	No effect described
850 (Serine)	2 (Gloeckner et al., 2010; Muda et al., 2014)	*In vitro* autokinase assay followed by mass spectrometric analysis Phosphopeptide enrichment by PKA followed by mass spectrometric analysis	Constitutive (Gloeckner et al., 2010) PKA phosphorylation (Muda et al., 2014)	No	No effect described
858 (Serine)	2 (Gloeckner et al., 2010; Muda et al., 2014)	*In vitro* autokinase assay followed by mass spectrometric analysis Phosphopeptide enrichment by PKA followed by mass spectrometric analysis	Constitutive (Gloeckner et al., 2010) PKA phosphorylation (Muda et al., 2014)	No	No effect described
860 (Serine)	3 (Gloeckner et al., 2010; Nichols et al., 2010; Muda et al., 2014)	*In vitro* autokinase assay followed by mass spectrometric analysis Purified LRRK2 from mammalian cell culture followed by mass spectrometer Phosphopeptide enrichment by PKA followed by mass spectrometric analysis	Constitutive (Gloeckner et al., 2010)	No	Phosphorylated by PKA (Muda et al., 2014)
865 (Serine)	1 (Gloeckner et al., 2010)	*In vitro* autokinase assay followed by mass spectrometric analysis	Constitutive (Gloeckner et al., 2010)	No	No effect described
895 (Serine)	1 (Gloeckner et al., 2010)	*In vitro* autokinase assay followed by mass spectrometric analysis	Constitutive (Gloeckner et al., 2010)	No	No effect described
898 (Serine)	1 (Gloeckner et al., 2010)	*In vitro* autokinase assay followed by mass spectrometric analysis	Constitutive (Gloeckner et al., 2010)	No	No effect described
908 (Serine)	2 (Gloeckner et al., 2010; Muda et al., 2014)	*In vitro* autokinase assay followed by mass spectrometric analysis Phosphopeptide enrichment by PKA followed by mass spectrometric analysis	Constitutive (Gloeckner et al., 2010) PKA phosphorylation (Muda et al., 2014)	No	No effect described
910 (Serine)	More than 5 (Nichols et al., 2010; Sheng et al., 2012; Muda et al., 2014)	Purified LRRK2 from mammalian cell culture followed by mass spectrometer	Constitutive (Gloeckner et al., 2010) PKA phosphorylation (Muda et al., 2014)	Yes Clone UDD1 15(3)	Many descriptions of this site, see description in text
912 (Serine)	2 (Gloeckner et al., 2010; Li et al., 2011)	*In vitro* autokinase assay followed by mass spectrometric analysis Affinity purification and mass spectrometric analysis from mouse brain	Constitutive (Gloeckner et al., 2010) From mice brains (Li et al., 2011)	No	No effect described
926 (Serine)	1 (Gloeckner et al., 2010)	*In vitro* autokinase assay followed by mass spectrometric analysis	Constitutive (Gloeckner et al., 2010)	No	No effect described
933 (Serine)	2 (Gloeckner et al., 2010; Muda et al., 2014)	*In vitro* autokinase assay followed by mass spectrometric analysis Phosphopeptide enrichment by PKA followed by mass spectrometric analysis	Constitutive (Gloeckner et al., 2010) PKA phosphorylation (Muda et al., 2014)	No	No effect described
935 (Serine)	More than 5 (Gloeckner et al., 2010; Muda et al., 2014)	Purified LRRK2 from mammalian cell culture followed by mass spectrometer	Constitutive (Gloeckner et al., 2010) PKA phosphorylation (Muda et al., 2014)	Yes Clone UDD2 10(12)	Many descriptions of this site, see description in text
954 (Serine)	2 (Gloeckner et al., 2010; Muda et al., 2014)	*In vitro* autokinase assay followed by mass spectrometric analysis Phosphopeptide enrichment by PKA followed by mass spectrometric analysis	Constitutive (Gloeckner et al., 2010) PKA phosphorylation (Muda et al., 2014)	No	No effect described
955 (Serine)	More than 5 (Gloeckner et al., 2010; Muda et al., 2014)	*In vitro* autokinase assay followed by mass spectrometric analysis Phosphopeptide enrichment by PKA followed by mass spectrometric analysis	Constitutive (Gloeckner et al., 2010) PKA phosphorylation (Muda et al., 2014)	Yes Clone MJF-R11 (75-1)	Many descriptions of this site, see description in text
958 (Serine)	2 (Gloeckner et al., 2010; Muda et al., 2014)	*In vitro* autokinase assay followed by mass spectrometric analysis Phosphopeptide enrichment by PKA followed by mass spectrometric analysis	Constitutive (Gloeckner et al., 2010) PKA phosphorylation (Muda et al., 2014)	No	No effect described
962 (Serine)	1 (Muda et al., 2014)	Phosphopeptide enrichment by PKA followed by mass spectrometric analysis	PKA phosphorylation (Muda et al., 2014)	No	No effect described
971 (Serine)	2 (Gloeckner et al., 2010; Muda et al., 2014)	*In vitro* autokinase assay followed by mass spectrometric analysis Phosphopeptide enrichment by PKA followed by mass spectrometric analysis	Constitutive (Gloeckner et al., 2010) PKA phosphorylation (Muda et al., 2014)	No	No effect described
973 (Serine)	More than 5 (Gloeckner et al., 2010; Muda et al., 2014)	*In vitro* autokinase assay followed by mass spectrometric analysis Phosphopeptide enrichment by PKA followed by mass spectrometric analysis	Constitutive (Gloeckner et al., 2010) PKA phosphorylation (Muda et al., 2014)	Yes Clone MJF-R12 (37-1)	Many descriptions of this site, see description in text
975 (Serine)	1 (Gloeckner et al., 2010)	*In vitro* autokinase assay followed by mass spectrometric analysis	Constitutive (Gloeckner et al., 2010)	No	No effect described
976 (Serine)	More than 5 (Nichols et al., 2010; Doggett et al., 2012; Muda et al., 2014)	*In vitro* autokinase assay followed by mass spectrometric analysis Phosphopeptide enrichment by PKA followed by mass spectrometric analysis	Constitutive (Gloeckner et al., 2010) PKA phosphorylation (Muda et al., 2014)	No	Many descriptions of this site, see description in text
979 (Serine)	2 (Gloeckner et al., 2010; Muda et al., 2014)	*In vitro* autokinase assay followed by mass spectrometric analysis Phosphopeptide enrichment by PKA followed by mass spectrometric analysis	Constitutive (Gloeckner et al., 2010) PKA phosphorylation (Muda et al., 2014)	No	No effect described
1024 (Threonine)	1 (Greggio et al., 2009)	Tandem MS/MS on phospho-purified proteins	Potential phosphosite (Greggio et al., 2009)	No	No effect described
1025 (Serine)	1 (Greggio et al., 2009)	Tandem MS/MS on phospho-purified proteins	Potential phosphosite (Greggio et al., 2009)	No	No effect described
1124 (Serine)	1 (Gloeckner et al., 2010)	*In vitro* autokinase assay followed by mass spectrometric analysis	Autophosphorylation (Gloeckner et al., 2010)	No	No effect described
1253 (Serine)	1 (Pungaliya et al., 2010)	LRRK2 G2019S assayed for autophosphorylation followed by analysis by LC-MS/MS	Autophosphorylation (Pungaliya et al., 2010)	No	No effect described
1283 (Serine)	1 (Pungaliya et al., 2010)	LRRK2 G2019S assayed for autophosphorylation followed by analysis by LC-MS/MS	Autophosphorylation (Pungaliya et al., 2010)	No	No effect described
1292 (Serine)	More than 5 (Sheng et al., 2012; Reynolds et al., 2014; Melachroinou et al., 2016; Purlyte et al., 2018)	*In vitro* autokinase assay followed by mass spectrometric analysis LRRK2 G2019S assayed for autophosphorylation followed by analysis by LC-MS/MS	Autophosphorylation (Gloeckner et al., 2010) Autophosphorylation (Pungaliya et al., 2010)	Yes Clone MJFR-19-7-8	Many descriptions of this site, see description in text
1332 (Tyrosine)	1 (Pungaliya et al., 2010)	LRRK2 G2019S assayed for autophosphorylation followed by analysis by LC-MS/MS	Autophosphorylation (Pungaliya et al., 2010)	No	No effect described
1343 (Threonine)	More than 5 (Greggio et al., 2009; Gloeckner et al., 2010; Pungaliya et al., 2010; Webber et al., 2011; Sheng et al., 2012; Law et al., 2014)	*In vitro* autokinase assay followed by mass spectrometric analysis LRRK2 G2019S assayed for autophosphorylation followed by analysis by LC-MS/MS Tandem MS/MS on phospho-purified proteins	Autophosphorylation (Gloeckner et al., 2010) Autophosphorylation (Pungaliya et al., 2010) Potential Phosphosite (Greggio et al., 2009)	No	Molecular association, regulation. Mutant T1343G do not change the kinase activity (Deng et al., 2008)
1345 (Serine)	3 (Greggio et al., 2009; Gloeckner et al., 2010; Pungaliya et al., 2010)	*In vitro* autokinase assay followed by mass spectrometric analysis LRRK2 G2019S assayed for autophosphorylation followed by analysis by LC-MS/MS Tandem MS/MS on phospho-purified proteins	Autophosphorylation (Gloeckner et al., 2010) Autophosphorylation (Pungaliya et al., 2010) Potential Phosphosite (Greggio et al., 2009)	No	No effect described
1348 (Threonine)	More than 5 (Greggio et al., 2009; Gloeckner et al., 2010; Taymans et al., 2011; Webber et al., 2011; Sheng et al., 2012)	*In vitro* autokinase assay followed by mass spectrometric analysis	Autophosphorylation (Gloeckner et al., 2010) Potential Phosphosite (Greggio et al., 2009)	No	Mutant T1348N presents a strong reduction of GTPases activity (Ito et al., 2007; Taymans et al., 2011) Mutant T1348A and T1348D present a strong reduction of GTP binding and strong reduction of autophosphorylation (Kamikawaji et al., 2013)
1349 (Threonine)	1 (Greggio et al., 2009)	Tandem MS/MS on phospho-purified proteins	Potential Phosphosite (Greggio et al., 2009)	No	Mutant T1349D but not T1349A presents a strong reduction of GTP binding and strong reduction of autophosphorylation (Kamikawaji et al., 2013)
1357 (Threonine)	3 (Pungaliya et al., 2010; Webber et al., 2011; Sheng et al., 2012)	LRRK2 G2019S assayed for autophosphorylation followed by analysis by LC-MS/MS Purified LRRK2 followed by a LTQ Orbitrap XL mass spectrometer analysis Recombinant LRRK2 protein purified from HEK293FT analyzed by LTQ-FTICR LC-MS system	Autophosphorylation (Pungaliya et al., 2010)	Yes developed by Kamikawaji et al. (2013)	T1357A mutant shows a decreased kinase activity (Liu et al., 2016) T1357A and T1357D show a decreased kinase activity and GTP binding (Kamikawaji et al., 2013)
1368 (Threonine)	4 (Gloeckner et al., 2010; Pungaliya et al., 2010; Webber et al., 2011; Sheng et al., 2012)	*In vitro* autokinase assay followed by mass spectrometric analysis LRRK2 G2019S assayed for autophosphorylation followed by analysis by LC-MS/MS Purified LRRK2 followed by an LTQ Orbitrap XL mass spectrometer analysis Recombinant LRRK2 protein purified from HEK293FT analyzed by LTQ-FTICR LC-MS system	Autophosphorylation (Gloeckner et al., 2010) Autophosphorylation (Pungaliya et al., 2010)	No	No effect described
1402 (Tyrosine)	1 (Pungaliya et al., 2010)	LRRK2 G2019S assayed for autophosphorylation followed by analysis by LC-MS/MS	Autophosphorylation (Pungaliya et al., 2010)	No	No effect described
1403 (Serine)	3 (Greggio et al., 2009; Kamikawaji et al., 2009; Pungaliya et al., 2010)	LRRK2 G2019S assayed for autophosphorylation followed by analysis by LC-MS/MS GST-ΔN-LRRK2 purified from Sf9 followed by a MALDI-TOF/MS Analysis Tandem MS/MS on phospho-purified proteins	Autophosphorylation (Pungaliya et al., 2010) Autophosphorylation (Kamikawaji et al., 2009) Potential Phosphosite (Greggio et al., 2009)	No	No effect described
1404 (Threonine)	3 (Greggio et al., 2009; Kamikawaji et al., 2009; Pungaliya et al., 2010)	LRRK2 G2019S assayed for autophosphorylation followed by analysis by LC-MS/MS GST-ΔN-LRRK2 purified from Sf9 followed by a MALDI-TOF/MS Analysis Tandem MS/MS on phospho-purified proteins	Autophosphorylation (Pungaliya et al., 2010) Autophosphorylation (Kamikawaji et al., 2009) Potential Phosphosite (Greggio et al., 2009)	No	No effect described
1410 (Threonine)	More than 5 (Pungaliya et al., 2010; Ito et al., 2014; Mamais et al., 2014; Melachroinou et al., 2016)	LRRK2 G2019S assayed for autophosphorylation followed by analysis by LC-MS/MS	Autophosphorylation (Gloeckner et al., 2010) Autophosphorylation (Pungaliya et al., 2010) Autophosphorylation (Kamikawaji et al., 2009) Potential Phosphosite (Greggio et al., 2009)	Yes MJFR4-25-5	Mutant T1410M presents a higher kinase activity and it is proapoptotic (Refai et al., 2015)
1443 (Serine)	2 (Pungaliya et al., 2010; Muda et al., 2014)	LRRK2 G2019S assayed for autophosphorylation followed by analysis by LC-MS/MS Phosphopeptide enrichment by PKA followed by mass spectrometric analysis	Autophosphorylation (Pungaliya et al., 2010) PKA phosphorylation (Muda et al., 2014)	No	Phosphorylated by PKA (Beilina et al., 2014)
1444 (Serine)	2 (Pungaliya et al., 2010; Muda et al., 2014)	LRRK2 G2019S assayed for autophosphorylation followed by analysis by LC-MS/MS Phosphopeptide enrichment by PKA followed by mass spectrometric analysis	PKA phosphorylation (Muda et al., 2014) Autophosphorylation (Pungaliya et al., 2010)	No	Phosphorylated by PKA (Beilina et al., 2014)
1445 (Serine)	1 (Pungaliya et al., 2010)	LRRK2 G2019S assayed for autophosphorylation followed by analysis by LC-MS/MS	Autophosphorylation (Pungaliya et al., 2010)	No	No effect described
1452 (Threonine)	3 (Greggio et al., 2009; Gloeckner et al., 2010; Pungaliya et al., 2010)	*In vitro* autokinase assay followed by mass spectrometric analysis LRRK2 G2019S assayed for autophosphorylation followed by analysis by LC-MS/MS Tandem MS/MS on phospho-purified proteins	Autophosphorylation (Gloeckner et al., 2010) Autophosphorylation (Pungaliya et al., 2010) Potential Phosphosite (Greggio et al., 2009)	No	No effect described
1457 (Serine)	1 (Pungaliya et al., 2010)	LRRK2 G2019S assayed for autophosphorylation followed by analysis by LC-MS/MS	Autophosphorylation (Pungaliya et al., 2010)	No	No effect described
1467 (Serine)	1 (Pungaliya et al., 2010)	LRRK2 G2019S assayed for autophosphorylation followed by analysis by LC-MS/MS	Autophosphorylation (Pungaliya et al., 2010)	No	No effect described
1470 (threonine)	1 (Pungaliya et al., 2010)	LRRK2 G2019S assayed for autophosphorylation followed by analysis by LC-MS/MS	Autophosphorylation (Pungaliya et al., 2010)	No	No effect described
1485 (Tyrosine)	1 (Pungaliya et al., 2010)	LRRK2 G2019S assayed for autophosphorylation followed by analysis by LC-MS/MS	Autophosphorylation (Pungaliya et al., 2010)	No	No effect described
1491 (Threonine)	More than 5 (Greggio et al., 2009; Kamikawaji et al., 2009; Gloeckner et al., 2010; Pungaliya et al., 2010; Webber et al., 2011; Doggett et al., 2012; Ito et al., 2014; Law et al., 2014; Reynolds et al., 2014)	Tandem MS/MS on phospho-purified proteins *In vitro* autokinase assay followed by mass spectrometric analysis	Autophosphorylation (Gloeckner et al., 2010) Autophosphorylation (Pungaliya et al., 2010) Autophosphorylation (Kamikawaji et al., 2009) Potential Phosphosite (Greggio et al., 2009)	Yes MJFR5-88-3	Behave the same way as S1292 (Reynolds et al., 2014)
1503 (Threonine)	More than 5 (Greggio et al., 2009; Gloeckner et al., 2010; Pungaliya et al., 2010; Webber et al., 2011; Doggett et al., 2012; Ito et al., 2014; Liu et al., 2014; Reynolds et al., 2014)	Tandem MS/MS on phospho-purified proteins *In vitro* autokinase assay followed by mass spectrometric analysis	Autophosphorylation (Gloeckner et al., 2010) Autophosphorylation (Pungaliya et al., 2010) Potential Phosphosite (Greggio et al., 2009)	Yes Clone MJF-R6 (227-1α)	Phosphorylation on this site decreases after transfection of 14-3-3; this phenomenon is not found with the phosphomutant S935A (Lavalley et al., 2016). T1503A mutant results in a greatly decreased GTP-binding and kinase activity; T1503D reduces only the GTP binding (Webber et al., 2011)
1508 (Serine)	1 (Pungaliya et al., 2010)	LRRK2 G2019S assayed for autophosphorylation followed by analysis by LC-MS/MS	Autophosphorylation (Pungaliya et al., 2010)	No	No effect described
1536 (Serine)	1 (Pungaliya et al., 2010)	LRRK2 G2019S assayed for autophosphorylation followed by analysis by LC-MS/MS	Autophosphorylation (Pungaliya et al., 2010)	No	No effect described
1612 (Threonine)	1 (Pungaliya et al., 2010)	LRRK2 G2019S assayed for autophosphorylation followed by analysis by LC-MS/MS	Autophosphorylation (Pungaliya et al., 2010)	No	No effect described
1627 (Serine)	1 (Shu et al., 2016)	Use of 1627 phosphomutant and incorporation of ^32P^ATP	CdK5 phosphorylation (Shu et al., 2016)	No	Phosphorylation of S1627 by Cdk5 could activate the LRRK2 kinase (Shu et al., 2016)
1647 (Serine)	1 (Pungaliya et al., 2010)	LRRK2 G2019S assayed for autophosphorylation followed by analysis by LC-MS/MS	Autophosphorylation (Pungaliya et al., 2010)	No	No effect described
1849 (Threonine)	2 (Pungaliya et al., 2010; Muda et al., 2014)	LRRK2 G2019S assayed for autophosphorylation followed by analysis by LC-MS/MS Phosphopeptide enrichment by PKA followed by mass spectrometric analysis	Autophosphorylation (Pungaliya et al., 2010)	No	Phosphorylated by PKA (Muda et al., 2014)
1853 (Serine)	1 (Pungaliya et al., 2010)	LRRK2 G2019S assayed for autophosphorylation followed by analysis by LC-MS/MS	Autophosphorylation (Pungaliya et al., 2010)	No	No effect described
1912 (Threonine)	1 (Pungaliya et al., 2010)	LRRK2 G2019S assayed for autophosphorylation followed by analysis by LC-MS/MS	Autophosphorylation (Pungaliya et al., 2010)	No	No effect described
1913 (Serine)	1 (Pungaliya et al., 2010)	LRRK2 G2019S assayed for autophosphorylation followed by analysis by LC-MS/MS	Autophosphorylation (Pungaliya et al., 2010)	No	No effect described
1967 (Threonine)	2 (Kamikawaji et al., 2009; Pungaliya et al., 2010)	LRRK2 G2019S assayed for autophosphorylation followed by analysis by LC-MS/MS GST-ΔN-LRRK2 purified from Sf9 followed by a MALDI-TOF/MS Analysis	Autophosphorylation (Pungaliya et al., 2010) Autophosphorylation (Kamikawaji et al., 2009)	No	No effect described
1969 (Threonine)	2 (Kamikawaji et al., 2009; Pungaliya et al., 2010)	LRRK2 G2019S assayed for autophosphorylation followed by analysis by LC-MS/MS GST-ΔN-LRRK2 purified from Sf9 followed by a MALDI-TOF/MS Analysis	Autophosphorylation (Pungaliya et al., 2010) Autophosphorylation (Kamikawaji et al., 2009)	No	No effect described
2031 (Threonine)	More than 5 (Greggio et al., 2009; Li et al., 2010; Pungaliya et al., 2010; Doggett et al., 2012; Ito et al., 2014)	Tandem MS/MS on phospho-purified proteins LRRK2 G2019S assayed for autophosphorylation followed by analysis by LC-MS/MS	Autophosphorylation (Pungaliya et al., 2010) Potential Phosphosite (Greggio et al., 2009)	Yes, developed in Li et al. (2010)	Study of phosphomutant indicates no change of kinase activity and no cytotoxicity (Li et al., 2010)
2032 (Serine)	More than 5 (Greggio et al., 2008, 2009; Li et al., 2010, 2015; Pungaliya et al., 2010; Ito et al., 2014)	Tandem MS/MS on phospho-purified proteins LRRK2 G2019S assayed for autophosphorylation followed by analysis by LC-MS/MS	Autophosphorylation (Pungaliya et al., 2010) Potential Phosphosite (Greggio et al., 2009)	Yes, developed in Li et al. (2010)	Mutant T2032A shows a reduced kinase activity (Li et al., 2010)
2035 (Threonine)	More than 5 (Greggio et al., 2008; Li et al., 2010; Pungaliya et al., 2010; Ito et al., 2014)	LRRK2 G2019S assayed for autophosphorylation followed by analysis by LC-MS/MS	Autophosphorylation (Pungaliya et al., 2010)	Yes, developed in Li et al. (2010)	Mutant T2035A shows a reduced kinase activity (Ito et al., 2007; Li et al., 2010)
2166 (Serine)	1 (Muda et al., 2014)	Phosphopeptide enrichment by PKA followed by mass spectrometric analysis	PKA phosphorylation (Muda et al., 2014)	No	No effect described
2257 (Serine)	1 (Pungaliya et al., 2010)	LRRK2 G2019S assayed for autophosphorylation followed by analysis by LC-MS/MS	Autophosphorylation (Pungaliya et al., 2010)	No	No effect described
2483 (Threonine)	1 (Gloeckner et al., 2010)	*In vitro* autokinase assay followed by mass spectrometric analysis	Autophosphorylation (Gloeckner et al., 2010)	Yes MJF-R8(21-2e)	Behave the same way as S1292 (Reynolds et al., 2014)
2524 (Threonine)	1 (Pungaliya et al., 2010)	LRRK2 G2019S assayed for autophosphorylation followed by analysis by LC-MS/MS	Autophosphorylation (Pungaliya et al., 2010)	No	No effect described

*Description of phosphorylation sites on human LRRK2. The first column describes the position of the phosphorylation site and the amino acid. The second column presents the number of paper using discovery mass spectrometry for LRRK2 phosphorylation sites. The third column shows the techniques used for the discovery of the phosphorylation site. The fourth column presents the nature of the phosphorylation site, auto- or heterologous phosphorylation site. The fifth column gives the name of the clone name of the antibody developed for the phosphorylation site. The last column gives information about the site and the effect associated to the phosphorylation*.

As is the case for many kinases, LRRK2 can autophosphorylate, and as for any phosphorylated kinase, it is therefore possible to divide the phosphorylated amino acids into two groups, the heterologous phosphorylation sites and the autophosphorylation sites. In addition, one can distinguish phosphosites that are observed from LRRK2 directly isolated from cells or tissues without any further manipulations (cellular phosphorylation sites) from sites that are submitted to additional *in vitro* phosphorylation. Sites that are qualified as autophosphorylation sites are confirmed when their phosphorylation rates increase after *in vitro* phosphorylation, while this is not the case for heterologous phosphorylation sites. Using these criteria, 60% of the identified LRRK2 phosphorylation sites are autophosphorylation sites and 36% are heterologous, while the remaining 4% of sites have been identified as both autophosphorylation and PKA phosphorylation sites (threonine 833, serine 1443, and serine 1444) (Gloeckner et al., 2010; Pungaliya et al., 2010; Muda et al., 2014).

Looking at the distribution of the phosphorylated residues across the LRRK2 protein, one prominent phosphorylation cluster is located between the ANK and the LRR domain at serines S860, S910, S935, S955, S973, and S976 for the most studied sites. The importance of the heterologous phosphorylation sites for LRRK2 function has been supported by the findings that 14-3-3 binding to LRRK2 is dependent on S910 and S935 phosphorylation and that LRRK2 phosphorylation levels at heterologous phosphorylation sites affect subcellular distribution of LRRK2 (see below).

The LRRK2 autophosphorylation occurs on at least 20 different serine or threonine residues located in and around the ROC domain and some in the kinase domain. While *in vitro* phosphorylation has revealed a large number of autophosphorylation sites, it remains unclear which proportion of these exist under physiological conditions. One example of an autophosphorylation site identified in cells and *in vivo* is the S1292 site that is positively modulated in LRRK2 mutants (N1437H, R1441C/G/H, Y1699C, G2019S, and I2020T) (Reynolds et al., 2014; Steger et al., 2016) as well as in rat brain lysate of BAC transgenic G2019S mice (Sheng et al., 2012). More recently, enhanced S1292 phosphorylation has also been identified in the brain, kidney, and lungs of LRRK2 G2019S knock-in (KI) mice (Kluss et al., 2018). The dephosphorylation of S1292 can be achieved *in cellulo*, by kinase inhibitors and evidence points to a role for protein phosphatase 2A (PP2A) in S1292 dephosphorylation (Reynolds et al., 2014).

## LRRK2 Phosphorylation Partners

**Figure 2:**
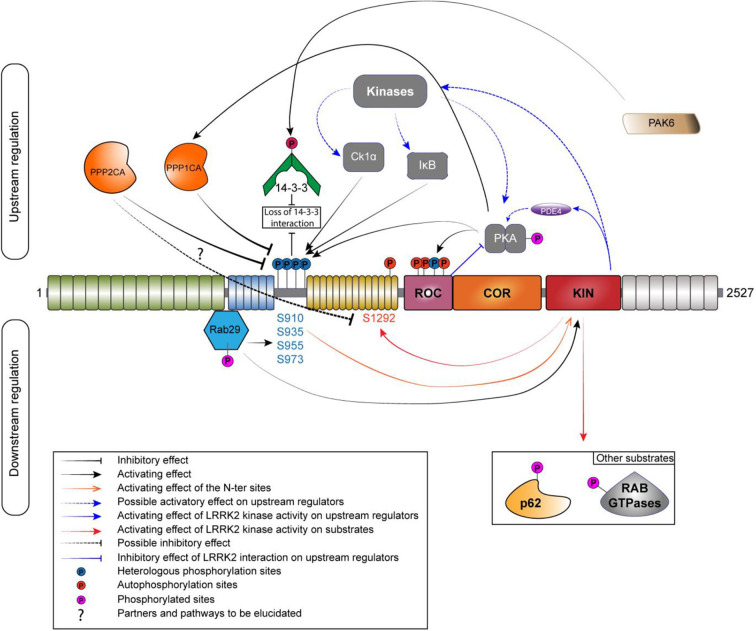
Phosphoregulation of LRRK2. Phosphoregulation of LRRK2 protein put together a lot of different partners, and some of those partners can also be regulated by LRRK2 itself. On the upstream regulation, the inhibitory phosphatases are localized on the left and the activating kinases are localized on the right. Kinases and phosphatases are implicated in the regulation of the N-ter phosphorylation sites (S910/935/955/973). N-ter sites and S1444 are phosphorylated by PKA while LRRK2 is also able to regulate the activity of PKA by a direct interaction with its ROC domain or by an indirect manner, by acting on the phosphodiesterase 4 (PDE4). PPP1CA has been confirmed to act on LRRK2. The holoenzyme PP2A could regulate the phosphorylation at S1292. The phosphorylation of the N-ter sites allows the interaction with 14-3-3. If phosphorylated by PAK6, the binding to LRRK2 is abolished. RAB29 interacts with LRRK2 in the Trans-Golgi network; this interaction leads to an increased phosphorylation of the N-ter and the kinase activity of LRRK2. LRRK2 can phosphorylate RAB29 and avoid LRRK2 activation, creating an inactivation loop (Nichols et al., 2010; Li et al., 2011; Dzamko et al., 2012; Lobbestael et al., 2013; Chia et al., 2014; Steger et al., 2016; Purlyte et al., 2018).

Protein phosphorylation is a key mechanism regulating protein function. This process is catalyzed by enzymes known as protein kinases, while the reverse reaction is mediated by protein phosphatases (Cohen, 2002; Manning et al., 2002). The balance of LRRK2 phosphorylation seems to be an element participating in the regulation of several cellular functions, including its cellular distribution (Nichols et al., 2010; Blanca Ramírez et al., 2017). Hence, elucidating the players involved in the regulation of LRRK2 phosphorylation balance (Figure 2) will be crucial to the understanding of how LRRK2 is (de)-regulated and affects downstream signaling processes. In addition to kinases and phosphatases, other cellular partners of LRRK2 contribute to determining LRRK2's phosphorylation status.

### LRRK2 Kinases

The first kinase reported as a candidate to regulate LRRK2 phosphorylation was PKA in 2007 (Ito et al., 2007). The authors of the study were able to identify PKA as an upstream kinase responsible for the phosphorylation of LRRK2 in HEK293 cells using two different potent inhibitors of PKA. They also showed that PKA efficiently phosphorylate LRRK2 K1906M kinase-inactive mutant. Several years later, two independent groups confirmed PKA as a kinase acting on the S910 and S935 sites (Li et al., 2011; Muda et al., 2014) and also on the S1444 site (Muda et al., 2014) both *in vitro* and *in cellulo*. Treatment with PKA activator forskolin increased phosphorylation at S910 as well as at S1444. The S1444 phosphorylation site was proposed as a new alternate 14-3-3 binding phosphosite. However, another study showed an opposite effect of PKA activation on LRRK2, with a decrease of phosphorylation at S910, S935, S955, and S973 and reduced 14-3-3 binding on LRRK2 overexpressed in HEK293 T-Rex cells and endogenous LRRK2 in A549 lung-derived cell lines (Reynolds et al., 2014). Finally, PKA activation or inhibition had no effect on the level of phosphorylation at pS935 (Hermanson et al., 2012). Further work will be needed to better decipher the role of PKA in the regulation of LRRK2 phosphorylation. Interestingly, the recent literature supports the notion of a functional cross-regulation between LRRK2 and PKA (reviewed in Greggio et al., 2017) that might be cell type specific (Parisiadou et al., 2014; Russo et al., 2018).

Dzamko et al. showed that the inhibitor of Ikappa B kinases (IKKα and β) phosphorylates the S910 and S935 sites in macrophages derived from bone marrow during stimulation of Toll-like receptor signaling (Dzamko et al., 2012). Further data indicate that IKKβ is also a potential kinase regulating LRRK2 phosphorylation in SH-SY5Y and HEK293 cells (Hermanson et al., 2012). Intriguingly, LRRK2 dephosphorylation induced by kinase inhibition with LRRK2-IN1 and CZC25146 was completely prevented by LPS stimulation (Dzamko et al., 2012).

Finally, Chia et al. provided the evidence that Casein Kinase 1-alpha (CK1α) is a physiologically upstream kinase regulator of LRRK2 at the constitutive phosphorylation sites using an unbiased siRNA kinome screen in HEK-293T cells as well as in the mouse brain with *ex vivo* experiment (Chia et al., 2014). In addition, the repression of the expression or inhibition of CK1α led to a decrease in phosphorylation at S910 and S935 as well as an increase in the association of ARHGEF7 with LRRK2, which decreased GTP binding. Treatment with siRNAs targeting CK1α also reduced RAB29-dependent Golgi fragmentation caused by LRRK2, indicating that phosphorylation of heterologous LRRK2 sites modulates recruitment of LRRK2 within the TGN (Chia et al., 2014). A number of additional upstream kinases have been proposed (Lobbestael et al., 2012). Nevertheless, identifying physiological kinase remain challenging but new advances (i.e., site-specific phospho-antibodies) will help to sort out true physiological upstream kinases regulating LRRK2 phosphorylation (list of specific antibodies described in Table 1).

### LRRK2 Phosphatases

The rapid induction of LRRK2 dephosphorylation after LRRK2 kinase inhibition suggests the involvement of protein phosphatases. Moreover, cAMP stimulation downregulated LRRK2 phosphorylation that suggests that a phosphatase may be activated in HEK293 but also in A549 cells (Hermanson et al., 2012).

The search of phosphatases related to LRRK2 pathophysiology has seen some advances in recent years. Regarding phosphatases regulating heterologous phosphorylation sites, only the alpha catalytic subunit of Protein Phosphatase 1 (PPP1CA) has been demonstrated to regulate phosphorylation of LRRK2 at S910, S935, S955, and S973 (Lobbestael et al., 2013). Indeed, pharmacological inhibition of Protein Phosphatase 1 (PP1) with Calyculin A (CalA) prevented the dephosphorylation of LRRK2 induced by LRRK2 kinase inhibitors. Interestingly, the effects of PPP1CA on LRRK2 phosphorylation were confirmed in several cell types HEK-293T, SH-SY5Y, NIH 3T3, A549, and U-2 OS but also in mouse primary cortical neurons. This shows that PPP1CA is active as an LRRK2 phosphatase independent of the cell type tested. Moreover, under LRRK2 dephosphorylation conditions, the association between PP1 and LRRK2 is increased, for example: during treatment with LRRK2 kinase inhibitors or in the presence of LRRK2 mutants with low level of phosphorylation (Lobbestael et al., 2013). Furthermore, a study on LRRK2 and oxidative stress (Mamais et al., 2014) also highlighted the importance of the physiological role of PP1 in the dephosphorylation of LRRK2. Arsenite-mediated stress leads to a reduction in the phosphorylation of LRRK2 at S910 and S935 in cell culture, and this reduction is reversed by CalA treatment. In addition, CalA counteracted arsenite and H_2_O_2_-induced S935 dephosphorylation, but only arsenite induced an increase association of PPP1CA with LRRK2 (Mamais et al., 2014). PP1 target specificity is driven by the association of regulatory subunits (Bollen et al., 2010). We do not yet know which regulatory subunits form the active PP1 holoenzyme responsible for catalyzing LRRK2 dephosphorylation. Therefore, a key issue to understand how LRRK2 dephosphorylation is regulated is to identify the composition of the PP1 holoenzyme by identifying the LRRK2-specific subunits that form the active PP1 holoenzyme that acts on LRRK2.

It is unclear which phosphatases are regulating LRRK2 phosphosites outside the ANK-LRR interdomain region. However, PP2A has been identified as a partner interacting with LRRK2 (Athanasopoulos et al., 2016). This study reports that LRRK2 interacts with all three subunits of PP2A and that this is mediated by the ROC domain in cultured cells. This is consistent with the recent report by Sim and colleagues who identified in a Drosophila model the three components of PP2A that are required to form a functional holoenzyme, i.e., scaffolding, regulatory, and catalytic subunits, as a modulator of LRRK2 function. Although PP2A involvement in the dephosphorylation of LRRK2 heterologous sites has yet to be tested, Athanasopoulos and colleagues observed a protective effect of the pharmacological activation of PP2A by sodium selenate in cells expressing the LRRK2 R1441C variant. In addition, silencing of the catalytic subunit of PP2A by shRNA aggravated cell degeneration in SH-SY5Y cells expressing the LRRK2 R1441C variant as well as in cultured cortical neurons derived from G2019S overexpressing transgenic mice. Interestingly, relevance of PP2A as an LRRK2 phosphatase for the regulation of S1292 phosphorylation site could be demonstrated by pharmacological and genetic approaches in mutant LRRK2 flies (Sim et al., 2019). Pharmacological activation with either ceramide or fingolimod (FTY720) ameliorates their disease-associated phenotypes. In addition, under conditions of PP2A subunit overexpression, LRRK2 phosphorylation at S1292 was found reduced. This is consistent with a report demonstrating that S1292 dephosphorylation is mediated by phosphatases that are sensitive to CalA and okadaic acid (Reynolds et al., 2014).

### LRRK2 Interactors

The phosphorylation at S910 and S935 sites, as well as the S1444 site, has been shown to be responsible for the interaction of LRRK2 with 14-3-3 proteins (Dzamko et al., 2010; Nichols et al., 2010; Li et al., 2011; Muda et al., 2014). Indeed, phosphodead mutations (substitution of the serine residue for alanine) at S910 and S935, but not at S955 and S973, lead to a strong reduction of 14-3-3 binding (Doggett et al., 2012). Moreover, if 14-3-3 binding is blocked using difopein (dimeric fourteen-three-three peptide inhibitor), LRRK2 appears to be dephosphorylated at S910 and S935 (Fraser et al., 2013; Zhao et al., 2015). Therefore, it has been suggested that 14-3-3 interaction could protect against dephosphorylation at these two phosphorylation sites and influence the subcellular localization of LRRK2 in the cell (Nichols et al., 2010; Li et al., 2011). The absence of 14-3-3 binding to LRRK2 when S910 and S935 sites are dephosphorylated induces accumulations of LRRK2 in the cytoplasm of cells. Accumulation types include filamentous “skein-like” structures (Dzamko et al., 2010; Nichols et al., 2010; Reyniers et al., 2014) and/or punctate accumulations (Chia et al., 2014). Likewise, pathogenic mutants that exhibit a reduction in phosphorylation at S910 and S935 sites (N1437H, R1441C/G/H, Y1699C, I2020T, and the risk factor G2385R, but not the G2019S variant) display a similar loss of 14-3-3 binding and relocalization of LRRK2 to cytoplasmic accumulations pools and filamentous skein-like structures (Dzamko et al., 2010; Nichols et al., 2010; Deng et al., 2011; Doggett et al., 2012). The brain is the tissue with the highest 14-3-3 concentration (Boston et al., 1982). The role of 14-3-3 proteins in neurodegeneration has been reviewed in Shimada et al. (2013) and is known to affect protein localization and activity through its binding to targeted substrates. Interestingly, there is an additional layer of regulation of 14-3-3 proteins that affects LRRK2 phosphorylation. Indeed, 14-3-3γ is phosphorylated by PAK6 (kinase 6 activated by p21), a serine/threonine kinase (Civiero et al., 2017). Phosphorylated 14-3-3γ is no longer able to bind S935 site, thus causing its dephosphorylation.

Several teams have demonstrated an interaction of LRRK2 with RAB29 (MacLeod et al., 2013; Beilina et al., 2014; Liu et al., 2017; Purlyte et al., 2018). This interaction takes place in the ANK domain of LRRK2 and regulates the heterologous phosphorylation sites of the S935 cluster (Purlyte et al., 2018), Purlyte et al. discovered that all RAB29 binding-deficient ankyrin domain LRRK2 variants are also dephosphorylated on these heterologous phosphorylation sites. In addition, the loss of endogenous RAB29 in A549 cells moderately reduces the phosphorylation of these sites. However, these data do not exclude the possibility that another Golgi resident, a protein kinase or phosphatase, regulates the phosphorylation of these sites. The LRRK2 kinase activity seems also to be regulated by RAB29 through the phosphorylation of the S935 cluster. In fact, the kinase activity of LRRK2 is reduced when a phosphomimetic mutant of RAB29 is expressed but no change is found with the dephosphomimetic form of RAB29. In particular, RAB29 is itself phosphorylated by LRRK2, suggesting that RAB29 binding to LRRK2 may mediate a potential positive feedback loop between LRRK2 phosphorylation at the S935 cluster and LRRK2 kinase activity, although further work would be required to confirm this (Purlyte et al., 2018). LRRK2 has other RABs as substrate but none of these have yet been reported to increase LRRK2's kinase activity.

A schematic of the relationship between LRRK2 and its different partners involved in its phosphoregulation is depicted in Figure 2. In addition, Table 2 provides an overview of the tissular distribution of the expression of LRRK2 with some of its primary regulators. This table indicates that, by and large, the LRRK2 phosphoregulators that have been studied in experimental systems are expressed in the same tissues as LRRK2, consistent with their potential physiological involvement in regulating LRRK2 in these tissues.

**Table 2:** Phosphoregulators of LRRK2 and RNA distribution in different tissues.

**Phosphoregulator of LRRK2**	**Brain**	**Lung**	**GI tract**	**Liver**	**Kidney**	**Muscle**	**Gonads**	**Skin**	**Blood**
LRRK2	+ (NX 1.4–5)	+++ (NX 50.4)	+ (NX 0.7–4.5)	+ (NX 4.5)	+ (NX 10.5)	+ (NX 4–6.7)	+ (NX 5.8–1.2)	+ (NX 2.6)	+/++ (NX 0–40.4)
PPP1CA	+/++ (NX 17.1–33.4)	++ (NX 27.6)	++ (NX 33.9–47.3)	+++ (NX 55)	++ (NX 43.7)	++ (NX 20.5–39)	+ (NX 20.1–15.7)	++ (NX 34.4)	+++ (NX 60–177)
PPP2CA	++ (NX 18.4–56.6)	++ (NX 22.7)	++ (NX 21.8–27.3)	++ (NX 33.2)	++ (NX 31.7)	++ (NX 31.6–47.9)	+/++ (NX 18.4–23.4)	++ (NX 32.6)	++ (NX 29.2–43.8)
PAK6	+ (NX 0.3–20.1)	+ (NX 2.6)	+ (NX 1.2–3.6)	+ (NX 0.4)	+ (NX 3.4)	+ (NX 0.8–7.9)	+ (NX 0.6–12.8)	++ (NX 22.2)	+ (NX 0.7–5.5)
IKKB	+/++ (NX 6.9–26.1)	+ (NX 14.9)	+ (NX 4.7–10.9)	+ (NX 9.9)	+ (NX 12.4)	+ (NX 7.9–17.6)	+ (NX 9.6–8.3)	++ (NX 22.2)	+ (NX 5.8–14.8)
CK1α	++ (NX 24.1–39.3)	++ (NX 37.5)	++ (NX 23.2–38.2)	++ (NX 30.1)	++ (NX 24)	++/+++ (NX 45.7–51.4)	++ (NX 28.6–31.7)	++ (NX 48.4)	++ (NX 28.6–47.4)
PKARIIβ	+/++/+++ (NX 4.9–85.5)	+ (NX 6.9)	+/++ (NX 4.8–23.6)	+ (NX 9)	+ (NX 10.6)	+ (NX 3.2–12.6)	+ (NX 3.6–8.3)	+ (NX 2.6)	+/++ (NX 5.4–27.6)
RAB29	+ (NX 5.9–20.2)	+ (NX 9.3)	+ (NX 6.8–10.4)	+ (NX 19.9)	++ (NX 46.6)	+ (NX 5.2–13)	+ (NX 3.2–5.8)	+ (NX 4.4)	+ (NX 11.6–34.8)

*RNA expression present data from RNA-seq from the Genotype-Tissue Expression project, FANTOM5 project, and CAGE data. NX, for Normalized eXpression has been calculated from the three transcriptomics datasets. Results presented in this table are the consensus transcript expression; here, the NX value represents the maximum NX value in the three data sources. Expression of mRNA is colored from light gray for low expression to black for high expression. Low = < 20 NX (+); Medium = 20–50 NX (++); High = >50 NX (+++)*.

### Other Regulators of LRRK2 Phosphorylation

In addition to cellular partners, other conditions regulating LRRK2 phosphorylation have been reported such as pharmacological agents or conditions in the cellular environment. Some of the strongest effects on LRRK2 phosphorylation are observed after treatment with LRRK2 pharmacological kinase inhibitors, either in cells or *in vivo*. Indeed, pharmacological inhibition of the LRRK2 kinase function leads to dephosphorylation of LRRK2 at the S935 cluster and loss of 14-3-3 binding (Dzamko et al., 2010; Deng et al., 2011). It is interesting to note that the S935 dephosphorylation of LRRK2 is observed both after treatment with LRRK2 kinase inhibitors that are considered potential therapeutics for PD and are currently in clinical testing (Doggett et al., 2012; Ding and Ren, 2020), as well as for several disease mutant forms of LRRK2. In both cases, the dephosphorylation can be explained by the recruitment of PPP1CA to the LRRK2 complex (Lobbestael et al., 2013). Nevertheless, further work will be needed to explain how LRRK2 dephosphorylation can be associated with, on the one hand, cellular toxicity (expression of LRRK2 disease mutants) and, on the other hand, the alleviation of cellular toxicity (for pharmacological inhibition of LRRK2). The dephosphorylation of LRRK2 at the S935 cluster after pharmacological treatment with LRRK2 kinase inhibitors in cells and in animal models is very strong and directly related to *in vitro* kinase inhibition (Dzamko et al., 2010; Vancraenenbroeck et al., 2014). It is therefore considered a pharmacodynamic marker for biological activity of LRRK2 kinase inhibitors (Fell et al., 2015; Taymans and Greggio, 2016). Besides effects on S935, pharmacological LRRK2 kinase inhibition also leads to dephosphorylation at the S1292 autophosphorylation site, providing a second pharmacodynamic readout (Sheng et al., 2012). In addition to LRRK2 kinase inhibitors, other treatments that are reported to affect LRRK2 phosphorylation are oxidative stress or activation of immune pathways (see above).

LRRK2 phosphorylation has also been measured for functional mutants of LRRK2. Mutants inhibiting GTP binding of LRRK2 (K1347A, T1348N) show a dephosphorylation of LRRK2, suggesting that phosphorylation of LRRK2 depends on its GTP-binding activity (Ito et al., 2007; Taymans et al., 2011). By contrast, while several functional mutants of LRRK2 that affect its kinase activity (either by inhibiting kinase activity or by activating kinase activity) have been observed to affect LRRK2 phosphorylation at the S935 cluster, there is no correlation between LRRK2 kinase activity and LRRK2 S935 phosphorylation (Ito et al., 2014; Reynolds et al., 2014). For instance, kinase dead mutants of LRRK2 are observed to either have no effect on LRRK2 S935 phosphorylation (such as for the K1906M mutant) or lead to a dephosphorylation (Ito et al., 2014; Reynolds et al., 2014). Phosphorylation phenotypes of LRRK2 mutants are summarized in Table 3.

**Table 3:** Overview of LRRK2 mutations that affect LRRK2 kinase inactivity. List of described mutation of LRRK2.

**Mutation**	**Familial/*in vitro* mutation**	**Cell type/*in vitro* assay**	**910**	**935**	**955**	**Others**
K1906A	*In vitro* mutation	HEK293T	Similar to WT (Ito et al., 2014)	Similar to WT (Ito et al., 2014)	Similar to WT (Ito et al., 2014)	No pThr1357/1491/1503 (Ito et al., 2014)
K1906M	*In vitro* mutation	HEK293T	Similar to WT (Ito et al., 2014)	Similar to WT (Ito et al., 2014)	Similar to WT (Ito et al., 2014)	No pThr1357/1491/1503 (Ito et al., 2014)
D1994A	*In vitro* mutation	HEK293T	Strong reduction (Ito et al., 2014)	Strong reduction (Ito et al., 2014)	Strong reduction (Ito et al., 2014)	No pThr1357/1491/1503 (Ito et al., 2014)
D1994N	*In vitro* mutation	HEK293T	Strong reduction (Ito et al., 2014)	Strong reduction (Ito et al., 2014)	Strong reduction (Ito et al., 2014)	No pThr1357/1491/1503 (Ito et al., 2014)
D2017A	*In vitro* mutation	HEK293T	Slight reduction (Ito et al., 2014)	No change (Ito et al., 2014)	No change (Ito et al., 2014)	No pThr1357/1491/1503 (Ito et al., 2014)
S2032A	*In vitro* mutation	HEK293T	No change (Ito et al., 2014)	No change (Ito et al., 2014)	No change (Ito et al., 2014)	No information
T2035A	*In vitro* mutation	HEK293T	No change (Ito et al., 2014)	No change (Ito et al., 2014)	No change (Ito et al., 2014)	No pThr1357/1491/1503 (Ito et al., 2014)
I2020T	*In vitro* mutation	HEK293T	Strong reduction (Doggett et al., 2012)	Strong reduction (Doggett et al., 2012)	Strong reduction (Doggett et al., 2012)	Increased S1292 (Kluss et al., 2018)
G2019S	*In vitro* mutation	HEK293T	No change (Ito et al., 2014)	No change (Ito et al., 2014)	No change (Ito et al., 2014)	Increased pT1491 (Ito et al., 2014)
T2031S	*In vitro* mutation	HEK293T	No change (Ito et al., 2014)	No change (Ito et al., 2014)	No change (Ito et al., 2014)	Increased P32 incorporation compared to WT (Nichols et al., 2010)
Y2018F	*In vitro* mutation	No test in cell line, *in vitro* assay	No change (Schmidt et al., 2019)	No change (Schmidt et al., 2019)	No change (Schmidt et al., 2019)	No phosphorylation of T1491 (Schmidt et al., 2019)
A2016T	*In vitro* mutation	HEK293T	Strong reduction (Ito et al., 2014)	Strong reduction (Ito et al., 2014)	Strong reduction (Ito et al., 2014)	No information

*Mutation presented in this table affect the phosphorylation of LRRK2. Their location and phosphorylation changes expressed in a particular cell type are presented and changes in phosphorylation are presented for Serine 910, 935, 955, and others*.

## Phenotypes and Pathomechanisms of LRRK2 Phosphorylation

While the global picture of how LRRK2 phosphorylation levels at its various phosphorylation sites influence LRRK2 function is still incomplete, several studies have shown that changes in LRRK2 phosphorylation influences LRRK2 biochemical or cellular properties and can be correlated to changes observed in PD patients and PD models. One key question pertaining to the effects of LRRK2 phosphorylation is how the LRRK2 phosphorylation status affects physiological and pathological mechanisms in PD and disease models. Evidence suggesting a correlation between LRRK2 phosphorylation and disease is growing, but much remains to be elucidated. Links between LRRK2 phosphorylation and disease or pathological mechanisms are being established in different ways: by monitoring LRRK2 phosphorylation in patient-derived samples, disease models, and study of phosphomutant forms of LRRK2 and how these affect cellular phenotypes.

### Phenotypic Correlates of LRRK2 Phosphorylation in PD Patients

Phosphorylation of the ANK-LRR cluster (S910, S935, S973) is found to be dephosphorylated in the substantia nigra of sporadic PD patients. S935 is also dephosphorylated in the amygdala and frontal cortex of PD patients. Immunostaining of brain tissues shows a high proportion of LRRK2 in neurons (Dzamko et al., 2017). By proximity ligation assay in dopaminergic neurons, Di Maio et al. show that the reduction in S935 phosphorylation is accompanied by an increase of S1292 (Di Maio et al., 2018). Interestingly, the phosphorylation of LRRK2 at S1292 is higher in SNCA^−/−^ HEK293 cells when oligomeric but not monomeric alpha-synuclein (α-syn) is present, suggesting a link between LRRK2 S1292 phosphorylation and species of α-syn linked to pathology that could be found in PD patient-derived cells.

Other hints to how phosphorylation levels of LRRK2 are correlated to PD come from studies in human biofluids. LRRK2 protein is secreted in exosomes of different biofluids in humans, including CSF and urine (Fraser et al., 2013). Studies in clinical cohorts report that pS1292-LRRK2 levels are elevated in urinary exosomes from G2019S LRRK2 mutation carriers compared to non-carriers and that PD manifesting G2019S LRRK2 mutation carriers have a higher S1292-LRRK2 level than the non-manifesting mutations carriers (Fraser et al., 2016a). The same group that performed a comparison between 79 PD patients and 79 healthy controls showed a higher level of pS1292-LRRK2 in PD urinary exosomes compared to healthy controls. In the same study, a correlation was established between the level of S1292 in urinary exosomes of idiopathic PD and cognitive impairment (Fraser et al., 2016b). A follow up study from the same group quantified higher levels of pS1292-LRRK2 in CSF exosomes compared to urinary exosomes, suggesting a higher LRRK2 kinase activity in the brain compared to that in the peripheral tissues (Wang et al., 2017). Further studies are now required to extend this work to include larger cohorts and assess reproducibility of the findings.

Finally, phospho-LRRK2 has also been measured in peripheral blood mononuclear cells (PBMCs) isolated from PD patients. A first report testing levels of S910 and S935 LRRK2 phosphorylation in idiopathic PD patients vs. controls found no variation of phosphorylation levels between the groups (Dzamko et al., 2013). However, when comparing individuals carrying the G2019S mutation with idiopathic PD patients, a significant reduction of S935-LRRK2 is observed (Padmanabhan et al., 2020). LRRK2 inhibitor treatment is also found to reduce the level of S935-LRRK2 in PBMCs (Delbroek et al., 2013) and in immortalized lymphocytes (Fernández et al., 2019). This dephosphorylation mediated by inhibitor acts also on S910, S955, and S973 on PBMCs from PD patients and controls (Perera et al., 2016). Therefore, LRRK2 phosphorylation in PBMCs holds promise to test for pharmacodynamic response in patients while further studies are required to ascertain whether LRRK2 measures in PBMCs have potential as disease biomarker.

### Phenotypic Correlates of LRRK2 Phosphorylation in PD Animal Models

The aim of this section is to identify whether changes in LRRK2 phosphorylation can be correlated to disease phenotypes in PD *in vivo* models. Therefore, we discuss studies in animal models of PD that have specifically measured LRRK2 phosphorylation levels. For a broader overview of PD animal models, we refer to previous review publications focusing on this subject (Blesa et al., 2012; Konnova and Swanberg, 2018). For instance, the systemic rotenone model, based on administration of the pesticide rotenone to rodents, mimics many aspects of PD. In this model, the phosphorylation of LRRK2 at S1292 is found to be increased in the microglia of rat substantia nigra after a rotenone treatment (Di Maio et al., 2018). Also, AAV-mediated α-syn overexpression in rats affects the phosphorylation of LRRK2 by increasing the phosphorylation of LRRK2 at S1292 in nigrostriatal dopamine neurons (Di Maio et al., 2018). There is a potential role for phosphatases in this finding as α-syn is reported, on the one hand, to positively regulate the activity of PP2A, a potential phosphoregulator of LRRK2 at S1292 (Reynolds et al., 2014) without affecting the protein level of PP2A in cell lines (Peng et al., 2005), but on the other hand, increased oligomerization and phosphorylation of α-syn reduced the activity of PP2A (Lou et al., 2010; Liu et al., 2015; Chen et al., 2016). Further research is needed to elucidate the link between the two important PD players LRRK2 and α-syn and how they influence each other's phosphorylation status (also reviewed in Taymans and Baekelandt, 2014).

Another study used the phosphomutant approach in a mouse model. The characterization of S910/S935 phosphorylation-deficient KI mice (i.e., where the serine has been replaced by an alanine, S910A/S935A) shows that they present a reduced phosphorylation of T73-RAB10 in the kidney, where LRRK2 is highly expressed, but no change in RAB10 phosphorylation in the brain. In terms of subcellular distribution of LRRK2, the S910A/S935A mice showed similar LRRK2 levels in the nuclear, chromatin bound and cytoskeletal fractions, but a significant decrease of the membrane-bound LRRK2 compared to the WT controls (Zhao et al., 2018). These mice showed signs of early PD dysfunction in their striatum including alterations in dopamine regulating proteins (decreases in tyrosine hydroxylase and dopamine transporter) and accumulation of α-synuclein, without degeneration of nigral dopaminergic neurons. Interestingly, these changes in dopamine regulating proteins are consistent with another study showing that LRRK2 phosphorylation levels are correlated with levodopa-induced dyskinesias in a rodent model (Stanic et al., 2016).

Studies in an LRRK2 G2019S KI transgenic mouse model have also correlated phenotypes to LRRK2 phosphorylation. Longo et al. investigate whether the KI of the G2019S LRRK2 mutation in mice causes functional changes in the neurons of the nigrostriatal system (Longo et al., 2017). Phenotypically, these mice reveal 63% increase in the dopamine uptake kinetics of maximal transport rate in the striatal synaptosomes compared to WT. In addition, the DAT protein level is 4-fold higher in G2019S KI compared to WT mice. Other studies on LRRK2 G2019S KI mice showed changes in vesicular physiology, notably with a reduction in basal and evoked dopamine in striata of aged mice (Yue et al., 2015) and an increase in glutamate release in cortical neuron culture derived from LRRK2 G2019S mice (Beccano-Kelly et al., 2014). The study also reveals that the LRRK2 phosphorylation level at S1292 is 8-fold higher in the striatum of 12-month-old G2019S KI mice compared to age-matched WT mice, confirming the gain of kinase activity of the G2019S mutation. The study also suggests that S1292 phosphorylation is correlated to changes in dopamine uptake. It remains to be determined whether S1292 phosphorylation itself mediates cell toxicity or if it is due to the change of kinase activity.

Levels of LRRK2 phosphorylation at the S935 cluster have also been monitored in bacterial artificial chromosome transgenic rats expressing LRRK2 mutants G2019S or R1441C. These rats display a significant impairment in motor function compared to the WT control rats (Sloan et al., 2016). Particularly, through the test of the accelerating rotarod, they showed that G2019S and R1441C rats between 18 and 21 months exhibit a significant age-dependent impaired performance compared to both non-transgenic and WT controls. Indeed at younger age, 3–6 months old, the transgenic rat lines showed no impairment on the rotarod test, whereas only G2019S rats showed an enhanced motor dysfunction, as previously reported (Zhou et al., 2011). Considering the increased importance of non-motor symptoms in PD, LRRK2 mutant rats have been analyzed for their cognitive ability, by using the spontaneous alternation test of spatial short-term memory. No differences in performance were seen in young adult G2019S or R1441C animals compared with controls. However, aged R1441C and G2019S rats showed significantly impaired performance on the spontaneous alternation test compared with WT controls. Interestingly, both rats show changes in LRRK2 phosphorylation but not in the same direction. LRRK2 G2019S transgenic rats show a modest increase in phosphorylation of the S935 site, while R1441C transgenic rats show dramatically reduced LRRK2 phosphorylation at residues S935 and S910 in the hippocampus. These data are consistent with the study from Nichols and colleagues showing reduced LRRK2 phosphorylation in R1441C KI mice (Nichols et al., 2010). Interestingly, the altered LRRK2 phosphorylation states, conversely to the motor impairment, is not age-dependent, appearing in both young and aged rats, suggesting that phosphorylation changes may be early markers of phenotypic changes. Further work is needed to explain differences in phosphorylation changes from mutant to mutant and how these changes contribute to the phenotypic consequences of the mutations.

The disease-causing mutation, G2019S-LRRK2 has also been associated with a decrease in arborization and neurite length in primary hippocampal and cortical cultures (Chan et al., 2011; Cho et al., 2013). Lavalley et al. (2016) observed a reversion of the neurite shortening caused by G2019S-LRRK2 expression in mouse model, via overexpression of 14-3-3, previously described as an important interactor of LRRK2 at different serine phosphosites: S910, S935 and S1444 (see section LRRK2 Interactors above). In this study, hemizygous 14-3-3θ-overexpressing mice were crossed with the BAC G2019S-LRRK2 transgenic mice and primary hippocampal cultures were prepared from pups at post-natal day 0. Primary cultures from the double transgenic mice show a reversed neurite shortening and an increase in LRRK2 phosphorylation at the S935 site. No effect on neurite length was detected in mice overexpressing 14-3-3θ alone compared to non-transgenic cultures. By contrast, PAK6-mediated 14-3-3 phosphorylation in neurons derived from LRRK2 G2019S mice, a condition that leads to LRRK2 dephosphorylation at S935, counteracts neurites shortening induced by the LRRK2 G2019S mutant (Civiero et al., 2017). While these studies show that modulation of LRRK2 phosphorylation (here for the S935 cluster) may alleviate negative effects of LRRK2 mutants in neurons and suggest a role for 14-3-3 proteins in the regulation of LRRK2-related toxicity, it remains unclear whether S935 dephosphorylation is detrimental, warranting further work on this question.

Besides rodent models, several studies have employed Drosophila models to study LRRK2 pathogenic mechanisms. Indeed, a study found that aged transgenic flies harboring G2019S or Y1699C LRRK2 variants exhibited DA neurodegeneration and concomitant locomotion deficits with a significant reduction in their climbing ability (Ng et al., 2009). Interestingly, Sim et al. identified through an unbiased RNAi-based phosphatase screen in the Drosophila LRRK2 G2019S mutant model that reduced expression of PP2A subunits in the flight muscles significantly delayed their locomotion ability in an age-dependent manner (Sim et al., 2019). This result proposed PP2A as a potential genetic modifier of LRRK2-induced toxicity. Intriguingly, they found that activation of PP2A mitigates dopaminergic dysfunction in this animal model as well as PP2A overexpression induced a reduction in LRRK2 phosphorylation at S1292, which was also reported by Reynolds et al. (2014). While these results remain to be confirmed in mammalian disease models, this study is consistent with the notion that the modulation of LRRK2 phosphorylation at S1292 via its phosphoregulators may affect pathological outcomes.

As a final note, measures of LRRK2 phosphorylation are regularly included in studies of PD animal models; therefore, including analysis of LRRK2 phosphorylation more systematically in future work in PD animal models is warranted.

### Mechanistic Comprehension

Regarding mechanisms of the phenotypes of LRRK2 phosphorylation, a first obvious question is whether LRRK2's phosphorylation status affects its own catalytic activity. To investigate the links between LRRK2 phosphorylation and its kinase activity, phosphomutants are used (effect on the LRRK2 phosphorylation are summarized in Table 4). When testing for autophosphorylation activity of the S910A/S935A mutant, no change in S1292 autophosphorylation was observed in cells compared to WT (Reynolds et al., 2014). Other phosphorylation site mutants or combinations of phosphorylation site mutants from the S935 cluster on LRRK2 kinase activity remain to be tested. By contrast, the effect of phosphorylation site mutants at LRRK2 autophosphorylation sites has been tested as summarized in Table 3. Mutant S2032A, T2035A, and S2032A/T2035A showed a reduced autophosphorylation activity, assessed by *in vitro* autophosphorylation with ^32^P-labeled ATP (Li et al., 2010). The overall conclusion here is that specific LRRK2 phosphorylation sites may affect LRRK2 kinase activity. Conversely, there is not a uniform correlation between LRRK2 phosphorylation and its kinase activity.

**Table 4:** Reported phosphosite mutants of LRRK2.

**Mutant**	**Phenotypic effect**
S910A	Induces the accumulation of LRRK2 in the cytoplasm of HEK293 cells with no change when treatment with inhibitor (puncta microtubules like structures) (Doggett et al., 2012) Results in loss of 14-3-3 interaction (Doggett et al., 2012)
S935A	Induces the accumulation of LRRK2 in the cytoplasm of HEK293 cells with no change when treatment with inhibitor (puncta microtubules like structures) (Doggett et al., 2012) Results in loss of 14-3-3 interaction (Doggett et al., 2012) The phosphorylation at T1503 does not decrease after transfection of 14-3-3 (Lavalley et al., 2016)
S955A	Localization is comparable to wild-type LRRK2 and presents the same pattern as WT when treated with LRRK2-IN1 (Doggett et al., 2012) No change of the kinase activity (P32 incorporation) (Reynolds et al., 2014)
S973A	Localization is comparable to wild-type LRRK2 and presents the same pattern as WT when treated with LRRK2-IN1 (Doggett et al., 2012) No change of the kinase activity (Reynolds et al., 2014)
S910A/935A	Induces the accumulation of LRRK2 in the cytoplasm of HEK293T. When treated with LRRK2-IN1, same pattern as LRRK2 WT treated with LRRK2-IN1 (Doggett et al., 2012) Results in loss of 14-3-3 interaction (Doggett et al., 2012) Does not increase the basal ubiquitination of LRRK2 (Zhao et al., 2015) No change of the kinase activity (P32 incorporation) (Reynolds et al., 2014)
S910/S935/S955/S973A	Trend to increased kinase activity (pSer1292 *p* = 0.066; pThr1491 *p* = 0.097; pThr2483 *p* = 0.055) (Reynolds et al., 2014)
S908A/S910A/S935A/S955A/S973A/S976A	LRRK2 is still capable of destabilization after kinase inhibitor treatment (MLi-2 or PF-06447475) (De Wit et al., 2019)
S908E/S910E/S935E/S955E/S973E/S976E	LRRK2 is still capable of destabilization after kinase inhibitor treatment (MLi-2 or PF-06447475) (De Wit et al., 2019)
S1292A	Reduced the percentage of cell with enlarged lysosomes (Henry et al., 2015) Reduced the effect of LRRK2 mutant on neurite growth of rats' cortical neurons (Sheng et al., 2012) Does not increase the basal ubiquitination of LRRK2 (Zhao et al., 2015) No change of the kinase activity (P32 incorporation) (Reynolds et al., 2014)
Thr1343A	Strong reduction of kinase activity (P32 incorporation) (Greggio et al., 2009) Decreased protein expression, suggesting destabilization of LRRK2 (Webber et al., 2011)
Thr1343A/1348A	No enzymatic activity (Liu et al., 2016)
Thr1348A	Mutant presents a strong reduction of GTP binding and strong reduction of kinase activity (P32 incorporation) (Kamikawaji et al., 2013) Strong reduction of kinase activity (P32 incorporation) (Greggio et al., 2009) Decreased protein expression, suggesting destabilization of LRRK2 (Webber et al., 2011)
Thr1348D	Mutant presents a strong reduction of GTP binding and strong reduction of kinase activity (P32 incorporation) (Kamikawaji et al., 2013)
Thr1349A	No change of GTP binding and kinase activity compared to WT (Kamikawaji et al., 2013) No increase kinase activity compared to WT LRRK2 (P32 incorporation) (Greggio et al., 2009)
Thr1349D	Reduced GTP binding and LRRK2 kinase activity (Kamikawaji et al., 2013)
Thr1357A	Mutant shows a decreased kinase activity (Liu et al., 2016) Mutant shows a decreased kinase activity and GTP binding (Kamikawaji et al., 2013).
Thr1357D	Mutant shows a decreased kinase activity and GTP binding (Kamikawaji et al., 2013)
S1403A	Increase of the kinase activity (P32 incorporation) (Greggio et al., 2009) No phosphorylation of Thr1491 autophosphorylation site (Kamikawaji et al., 2009)
Thr1404A	Increase of the kinase activity (P32 incorporation) (Greggio et al., 2009)
Thr1410D	Minor effect on the dimer formation compared to wild type (Pungaliya et al., 2010)
Thr1410A	No effect on dimer formation, and no effect on kinase activity on exogenous substrate. Reduction of GTPase activity without a reduction of binding to GTP (Pungaliya et al., 2010) Increase of the kinase activity (P32 incorporation) (Greggio et al., 2009) No phosphorylation of Thr1491 autophosphorylation site (Kamikawaji et al., 2009)
Thr1452A	Trend to decrease the kinase activity (P32 incorporation) (Greggio et al., 2009)
Thr1491A	No change of the kinase activity compared to WT (P32 incorporation) (Greggio et al., 2009) No change of the kinase activity (P32 incorporation) (Reynolds et al., 2014)
Thr1503A	Mutations led to decrease the proportion of LRRK2 bound to GTP and decrease the kinase activity (Webber et al., 2011) Slight increase of the kinase activity (P32 incorporation) (Greggio et al., 2009)
Thr1503D	Mutations led to decrease the proportion of LRRK2 bound to GTP, similarly to Thr1503A, without changing the kinase activity compared to WT LRRK2 (Webber et al., 2011).
Thr2031A	No effect on LRRK2 kinase activity, and no effect on LRRK2 toxicity (Li et al., 2010) No effect on LRRK2 kinase activity (Greggio et al., 2008)
Thr2031D	No effect on LRRK2 kinase activity, and no effect on LRRK2 toxicity (Li et al., 2010)
Thr2031E	No effect on LRRK2 kinase activity, and no effect on LRRK2 toxicity (Li et al., 2010)
S2032A	Reduced kinase activity (P32 incorporation) (Ito et al., 2014) Reduced kinase activity (P32 incorporation), and no effect on LRRK2 cytotoxicity (Li et al., 2010) Reduced kinase activity (P32 incorporation) (Greggio et al., 2008)
S2032D	No effect on LRRK2 toxicity (Li et al., 2010)
S2032E	No effect on LRRK2 toxicity (Li et al., 2010) No effect on kinase activity (P32 incorporation) (Greggio et al., 2008)
Thr2031A/Thr2032A	Modest cytotoxicity compared to pcDNA control (Li et al., 2010)
Thr2035A	Reduced kinase activity (P32 incorporation) and slightly rescued LRRK2 cytotoxicity (Li et al., 2010) Reduced kinase activity (P32 incorporation) (Greggio et al., 2008)
Thr2035D	Reduced kinase activity (P32 incorporation) and slightly rescued LRRK2 cytotoxicity (Li et al., 2010)
Thr2035E	Reduced kinase activity (P32 incorporation) and slightly rescued LRRK2 cytotoxicity (Li et al., 2010) Reduced kinase activity (P32 incorporation) (Greggio et al., 2008)
Thr2131A/S2032A	Reduced modestly the LRRK2 toxicity (Li et al., 2010)
S2031A/Thr2035A	Reduced kinase activity (P32 incorporation) and rescued LRRK2-induced toxicity (Li et al., 2010)
S2032A/Thr2035A	Reduced kinase activity (P32 incorporation) and rescued LRRK2-induced toxicity (Li et al., 2010)
Thr2031A/S2032A/Thr2035A	Reduces kinase activity (P32 incorporation) and completely attenuates cytotoxicity (Li et al., 2010)
Thr2483A	No change of the kinase activity (P32 incorporation) (Reynolds et al., 2014)

*List of LRRK2 phosphomutants followed by the description of the cellular phenotype associated to the phosphomutant. P32 incorporation is used here as in vitro autophosphorylation assay*.

Besides kinase activity, GTP-binding and GTPase activity may also be influenced by LRRK2 phosphorylation levels. Of particular interest are the autophosphorylation sites that are clustered in and around the ROC GTPase domain and several sites map to G-box motifs that mediate GTP binding, which point to the possibility that autophosphorylation may affect GTPase functions (Webber et al., 2011; Taymans, 2012). In particular, some phosphomimetic mutants such as T1491D and T1503D showed impaired GTP binding, although GTP binding is unchanged for another phosphomimetic LRRK2 mutant, T1410D (Kamikawaji et al., 2009; Webber et al., 2011). Further work is warranted to establish the precise link between autophosphorylation and LRRK2 GTP-binding and GTPase activity. By extension, a potential role of heterologous phosphorylation sites of LRRK2 on its GTP-related functions cannot be excluded. Nevertheless, these remain to be examined.

### Kinase Inhibition

As mentioned in section LRRK2 Phosphatases, pharmacological inhibition of LRRK2 kinase activity induces LRRK2 dephosphorylation. Moreover, the induction of LRRK2 ubiquitination has been observed after LRRK2 pharmacological kinase inhibition followed by decreased protein levels, due to proteasomal degradation (Zhao et al., 2015; Lobbestael et al., 2016). This suggests that one of the consequences of prolonged LRRK2 dephosphorylation at the S935 cluster may be LRRK2 degradation, although this effect may be tissue and condition specific. In rats, administration of LRRK2 kinase inhibitor PFE360 in food leads to a decrease of LRRK2 total protein level in the brain but not in lung (Kelly et al., 2018). In contrast, loss of LRRK2 protein level was not detected in mouse brain, consistent with results reported in other studies using MLi-2-treated mice (Fell et al., 2015). At the phenotypic level, LRRK2 kinase inhibitors induced abnormal cytoplasmic accumulation of secretory lysosome in the lungs but no change in the kidney in non-human primates (Fuji et al., 2015). Six hours of treatment with inhibitors of CK1α, an upstream kinase of the S935 cluster, induced dephosphorylation of S935 and protein destabilization. In fact, CK1α inhibition is able to destabilize LRRK2 mutant R1441G/I2020T and also mutant without ARM domain (De Wit et al., 2019).

While these data suggest the notion that LRRK2 dephosphorylation at S935 cluster may be a priming event for LRRK2 degradation, the reality of the mechanism is likely more complex. Loss of phosphorylation does not seem to be enough to destabilize the protein; LRRK2 dephosphomutant at six heterologous sites for S908A/910A/935A/955A/973A/976A does not show reduced basal expression levels, but this mutant is still degraded after 24 h of pharmacological inhibition in cell culture (De Wit et al., 2019). Other examples of discrepancies in LRRK2 expression in different conditions include KI mice for kinase dead variant of LRRK2, D1994S, that display decreased protein levels. However, those observations are not replicated in cells (Herzig et al., 2011). Also, R1441G and Y1699C mutants with low GTPase activity and reduced steady-state phosphorylation at the S935 cluster have an increased basal level of ubiquitination compared to the I2020T mutant that shows normal GTPase activity and increased kinase activity (De Wit et al., 2019). Pharmacological inhibition does not affect the ubiquitination level of those mutants and no destabilization is found after 48 h of kinase inhibitor treatment by MLi-2 or PFE-475. Ubiquitination level of those mutants can be restored with CalA. Intriguingly, the N-terminus sequence as well as S935 phosphosite of LRRK2 is involved in inhibitor-induced LRRK2 destabilization. Indeed, a truncated form of LRRK2 (170-kDa) that lacks the ARM domain is dephosphorylated on S1292 after kinase inhibition but not destabilized. Nevertheless, this version of LRRK2 does not present a phosphorylation at S935 (De Wit et al., 2019). Due to the LRRK2 protein destabilization observed in certain conditions after kinase inhibition, it should be noted that some of these phenotypes may correspond to phenotypes observed in LRRK2 KO animals. For instance, increased number and size of lysosomes in kidney proximal tubule cells and lamellar bodies in lung type II cells is found in LRRK2 KO mice (Herzig et al., 2011), while similar findings are made in LRRK2 KO rats (Baptista et al., 2013). Further research should be performed to further determine the hypothesized parallel between LRRK2 kinase inhibition and LRRK2 KO.

## Conclusions/Perspectives

Advances in the study of LRRK2 highlight the importance of LRRK2 phosphorylation both in its normal physiological function and, as far as the brain data suggest, in its pathological effects, warranting further investigation of the consequences of LRRK2 phosphoregulation on its functions. In particular, further study of LRRK2 phosphoregulation itself as well as the pursuit of efforts to correlate LRRK2 phosphorylation to phenotypes in cells, in *in vivo* PD models as well as in PD patients would be very valuable. The high number of phosphorylation sites in LRRK2 results in a complex image of the links between LRRK2 phosphorylation and the protein's behavior. A better understanding of the regulation of LRRK2 phosphorylation therefore might result in new perspectives for treatment and diagnosis of PD.

Box 1 **Box of outstanding issues.**

Based on the current state of our understanding of LRRK2 phosphorylation, several issues can be discerned:

- There is a need for an improved survey of LRRK2 phosphorylation sites in humans. LRRK2 phosphosites have often been discovered in experimental systems with LRRK2 overexpression. While some phosphosites have been confirmed under physiological conditions in cellular or *in vivo* models, relatively little has been done to confirm or detect new LRRK2 phosphosites in human tissues.
- In a similar fashion, the regulation of LRRK2 phosphorylation is regularly studied in overexpression conditions and there remains a need to confirm whether regulations found occur at the level of endogenously expressed proteins (both for LRRK2 and its phosphoregulators).
- While changes in LRRK2 phosphorylation levels have been described in disease, it remains an open question as to whether specific phosphorylation changes implicated in the disease mechanisms are biomarkers of disease or both. For instance, the finding that PD patients show S935-LRRK2 dephosphorylation in brain is in apparent contradiction to the same dephosphorylation induced by kinase inhibitors that are proposed as therapeutic agents in PD. Further work is needed to determine whether its level of phosphorylation is “healthy” or disease related and tissue/cell type specificity related.
- Related to this are apparent contradictions observed in model systems where phosphorylation changes in pathological conditions differ from one model to another. Such discrepant findings must be further explained in order to refine knowledge of what the phenotypes of LRRK2 phosphorylation are and develop better models to study consequences of LRRK2 phosphorylation changes.
- Advances in the mechanisms regulating LRRK2 phosphorylation have begun to reveal upstream kinases, phosphatases, and interaction partners involved in LRRK2 phosphoregulation and point to several instances of feedback mechanisms as well as interconnectedness between phosphoregulators. Further work is required to complete the list of LRRK2 phosphoregulators and fully elucidate the intricacies of the LRRK2 phosphoregulation complex.

## Author Contributions

AM and MD wrote the manuscript and designed the figures and tables. AS contributed text on the phenotypic models. J-MT and M-CC-H defined the scope of the manuscript and provided text contributions and edits. All authors reread and approved the manuscript.

## Conflict of Interest

The authors declare that the research was conducted in the absence of any commercial or financial relationships that could be construed as a potential conflict of interest.
